# Development of a clinically validated *in vitro* functional assay to assess pathogenicity of novel *GAA* variants in patients with Pompe disease identified *via* newborn screening

**DOI:** 10.3389/fgene.2022.1001154

**Published:** 2022-09-30

**Authors:** Shelly Goomber, Erin Huggins, Catherine W. Rehder, Jennifer L. Cohen, Deeksha S. Bali, Priya S. Kishnani

**Affiliations:** ^1^ Division of Medical Genetics, Department of Pediatrics, Duke University School of Medicine, Durham, NC, United States; ^2^ Department of Pathology, Duke University Medical Center, Durham, NC, United States; ^3^ Biochemical Genetics Laboratory, Duke University Medical Center, Durham, NC, United States

**Keywords:** pompe disease, newborn screening, variant classification, functional studies, *in vitro* assay, lysosomal storage disease (LSD), glycogen storage disease type 2

## Abstract

**Purpose:** The addition of Pompe disease (Glycogen Storage Disease Type II) to the Recommended Uniform Screening Panel in the United States has led to an increase in the number of variants of uncertain significance (VUS) and novel variants identified in the *GAA* gene. This presents a diagnostic challenge, especially in the setting of late-onset Pompe disease when symptoms are rarely apparent at birth. There is an unmet need for validated functional studies to aid in classification of *GAA* variants.

**Methods:** We developed an *in vitro* mammalian cell expression and functional analysis system based on guidelines established by the Clinical Genome Resource (ClinGen) Sequence Variant Interpretation Working Group for PS3/BS3. We validated the assay with 12 control variants and subsequently analyzed eight VUS or novel variants in *GAA* identified in patients with a positive newborn screen for Pompe disease without phenotypic evidence of infantile-onset disease.

**Results:** The control variants were analyzed in our expression system and an activity range was established. The pathogenic controls had GAA activity between 0% and 11% of normal. The benign or likely benign controls had an activity range of 54%–100%. The pseudodeficiency variant had activity of 17%. These ranges were then applied to the variants selected for functional studies. Using the threshold of <11%, we were able to apply PS3_ supporting to classify two variants as likely pathogenic (c.316C > T and c.1103G > A) and provide further evidence to support the classification of likely pathogenic for two variants (c.1721T > C and c.1048G > A). One variant (c.1123C > T) was able to be reclassified based on other supporting evidence. We were unable to reclassify three variants (c.664G > A, c.2450A > G, and c.1378G > A) due to insufficient or conflicting evidence.

**Conclusion:** We investigated eight *GAA* variants as proof of concept using our validated and reproducible *in vitro* expression and functional analysis system. While additional work is needed to further refine our system with additional controls and different variant types in order to apply the PS3/BS3 criteria at a higher level, this tool can be utilized for variant classification to meet the growing need for novel *GAA* variant classification in the era of newborn screening for Pompe disease.

## 1 Introduction

Pompe disease (OMIM 232300), also known as Glycogen Storage Disease Type II, is an autosomal recessive disorder caused by deficiency of the lysosomal enzyme acid alpha-1,4-glucosidase (GAA), leading to toxic accumulation of glycogen in cardiac, skeletal, and smooth muscle (Hers, 1963). This deficiency is caused by biallelic pathogenic variants in the *GAA* gene, which can affect transcription, protein expression, catalytic activity, or the intracellular trafficking of the translated protein, resulting in decreased functional GAA enzyme (Hers, 1963; Hermans et al., 1993; Moreland et al., 2005; Tager et al., 1987; Wisselaar et al., 1993). The disease ranges in severity from infantile-onset Pompe disease (IOPD), characterized by hypertrophic cardiomyopathy and rapidly progressing muscle weakness and death within the first 12 months of life, to late-onset Pompe disease (LOPD), a more slowly progressive form without cardiomyopathy in the first year of life and symptom onset varying across the lifespan (Kishnani et al., 2006). Currently, treatment is available across the full disease spectrum with enzyme replacement therapy (ERT).

Due to the availability of treatment and evidence from Taiwan’s newborn screening (NBS) program demonstrating improved clinical outcomes with early detection and treatment (Chien et al., 2009; Chien et al., 2011; Yang et al., 2016), Pompe disease was the first lysosomal storage disorder added to the Recommended Uniform Screening Panel (RUSP) in the United States in 2015. At the time of this publication, over half of US states screen for Pompe disease at birth. Low GAA enzyme activity in blood followed by *GAA* sequencing is accepted as the standard of care for clinical diagnostic testing (Chiang et al., 2012). Clinical decision making regarding follow up evaluation and treatment initiation is based on the results of these studies (Burton et al., 2017a). While there have been over 900 *GAA* variants described (Nino et al., 2019), many of which are variants of uncertain significance (VUS), the adoption of NBS has led to detection of increasing numbers of novel *GAA* variants and VUS (Burton et al., 2017b; Ficicioglu et al., 2020; Tang et al., 2020). At the time of this publication, there are 631 reported missense variants in *GAA* in ClinVar*;* 450 of them (71%) are classified as VUS and an additional 48 (7%) have conflicting interpretations of pathogenicity (Landrum et al., 2018). These numbers are rapidly increasing with the adoption of NBS. VUS and novel variant identification presents a challenge in the diagnosis and management of patients suspected to have Pompe disease, especially LOPD, in the setting of NBS.

Unlike patients with IOPD or clinically ascertained LOPD for whom diagnoses can be confirmed clinically and biochemically and treatment can be initiated in a timely manner, diagnosis of LOPD in the setting of NBS is more difficult. Infants with LOPD do not have cardiomyopathy, muscle weakness may be subtle, if present, and biomarkers such as urine glucose tetrasaccharide (Glc_4_), creatine kinase (CK), and aspartate aminotransferase (AST) are often normal in the pre-symptomatic phase of LOPD (Huggins et al., 2022). Thus, confirmation of a diagnosis of LOPD often relies heavily on *GAA* sequencing in the absence of a clear clinical phenotype. *GAA* sequencing is critical for the exclusion of carriers and/or those with pseudodeficiency alleles; this is especially challenging in the setting of one or more VUS or novel variants, which may occur in cis with a pseudodeficiency allele. Given that the majority (∼80%) of Pompe disease cases detected by NBS are ultimately classified as LOPD (Burton et al., 2017b), clear classification of *GAA* variants is crucial to the successful implementation of NBS. Unclear variant classification may lead to misdiagnosis, improper initiation of or delayed treatment with ERT, unnecessary healthcare expenses, and parent/caregiver anxiety. Thus, there is a time-sensitive need for proper classification of VUS and novel variants in the *GAA* gene.

Rare or novel missense *GAA* variants are challenging to classify without some evidence of the individual variant’s effect on GAA activity. In 2015, the American College of Medical Genetics and Genomics/Association for Molecular Pathology (ACMG/AMP) published guidelines for the interpretation of sequence variants (Richards et al., 2015). These guidelines established a robust framework for classification of sequence variants as likely pathogenic/pathogenic (LP/P) or likely benign/benign (LB/B) based on various types and strengths of available evidence (e.g., very strong, strong, moderate, and supporting). Variants that do not meet the threshold of evidence for LP or LB remain classified as a VUS. Well-validated *in vivo* and *in vitro* functional studies can provide strong evidence toward pathogenicity (PS3) or benign classification (BS3). These studies must be robust, reproducible, and informative for the specific variant type or disease mechanism being studied; however, the ACMG/AMP framework does not provide specific criteria for the validation of a functional assay that can be used as evidence for clinical variant classification. The Clinical Genome Resource (ClinGen) established the Sequence Variant Interpretation (SVI) Working Group^1^ with the goal of further refining the original criteria set forth by the ACMG/AMP guidance in 2015. The ClinGen SVI published specific recommendations for functional assay validation and the use of functional data in applying PS3/BS3 criteria towards classification of any variant (Brnich et al., 2019). The level of strength at which functional data can be applied is based on the number of validated controls used. In order for assays to meet a moderate level of evidence (PS3_moderate and BS3_moderate), a balanced mix of at least 11 well-established, known pathogenic and benign controls must be included in the assay validation. In order to meet a supporting level of evidence (PS3_supporting and BS3_supporting), fewer controls may be used to validate the assay with the number of pathogenic and the number of benign controls influencing the strength at which PS3 or BS3 may be applied.

Our team of clinical, molecular, and biochemical geneticists experienced in the diagnosis and management of Pompe disease developed a mammalian cell expression and functional analysis system with the above criteria in mind to evaluate the pathogenicity of VUS or novel variants detected in patients with a positive NBS for Pompe disease. Herein, we describe the development and validation of our *in vitro* functional assay using 12controls (7 pathogenic, 4 benign, and 1 pseudodeficiency variant) and describe the outcome of testing eight VUS or novel variants from seven unrelated patients with either suspected or confirmed LOPD identified *via* NBS.

## 2 Materials and methods

We developed an *in vitro* expression system using human embryonic kidney cells (HEK293). A combination of seven known pathogenic *GAA* variants (two null and five missense variants), four known benign/likely benign *GAA* variants (one silent change and three missense variants), and one known pseudodeficiency variant were selected as controls (*n* = 12). These controls were used to validate the functional assay and *in vitro* expression system (Table 1). The pathogenic control variants selected have been reported in classic IOPD when present in homozygosity, with the exception of c.670C >T which has been reported in childhood onset disease (de Faria et al., 2021; Nino et al., 2019). Eight variants identified in seven unrelated patients were selected for functional studies (Table 2). All patients had positive NBS for Pompe disease. Four patients (1–4) were enrolled in a Duke University Health System IRB-approved observational research study (Pro00010830, Pro00100223, or Pro00001562). Patients were classified as having LOPD based on deficient GAA enzyme activity in blood on confirmatory testing, and absence of cardiomyopathy within the first year of life. Three additional patients (5–7) were identified at external institutions and de-identified data were sent to our team to review. None of these patients harbored any known pseudodeficiency variants or alleles, which are known to suppress GAA activity levels *in vitro* enzyme assays. The functional impact of each selected variant was assessed using our validated *in vitro* expression system. Final variant classification was completed using results of the GAA functional analysis and additional information captured from publicly available databases and resources.

**TABLE 1:** Known pathogenic and benign variants used as functional assay controls. GAA enzyme activity expressed as % of wild-type detected for each known variant using the HEK293 cell-based assay.

Control variant	ClinVar accession	ClinVar classification	Residual GAA activity detected in our system
c.2560C >T (p.Arg854*)	VCV0000004034	Pathogenic	None detected
c.525delT (p.Glu176Argfs*45)	VCV000004033	Pathogenic	None detected
c.1933G >A (p.Asp645Asn)	VCV000188728	Pathogenic	None detected
c.655G >A (p.Gly219Arg)	VCV000189065	Pathogenic	11%
c.670C >T (p.Arg224Trp)	VCV000189188	Pathogenic	6.4%
c.925G >A (p.Gly309Arg)	VCV000188797	Pathogenic	1.3%
c.1655T >C (p.Leu552Pro)	VCV000279811	Pathogenic	7.8%
c.2338G >A (p.Val780Ile)	VCV000092476	Benign	100%
c.668G >A (p.Arg223His)	VCV000092488	Benign	100%
c.1935C >T (p.Asp645 = )	VCV001131864	Likely Benign	100%
c.596A >G (p.His199Arg)	VCV000092486	Benign	54%
c.1726G >A (p.Gly576Ser)	VCV000092467	Benign; other (Pseudodeficiency)	17%

**TABLE 2:** Genotypes of patients whose variants were selected for functional analysis. All patients had a confirmed pathogenic variant (Variant 1) except Patient 4.

Patient	Variant 1	Variant 2
1	c.−32-13T >G	c.316C >T, p.Arg106Cys
2	c.−32-13T >G	c.1103G >A, p.Gly368Asp
3	c.−32-13T >G	c.1721T >C, p.Leu574Pro
4	c.664G >A, p.Val222Met	c.2450A >G, p.His817Arg
5	c.1589del, p.Glu530Glyfs*48	c.1048G >A, p.Val350Met
6	c.−32-13T >G	c.1378G >A, p.Glu460Lys
7	c.−32-13T >G	c.1123C >T, p.Arg375Cys

### 2.1 Generation, expression, functional analysis and characterization of novel variants

pcDNA3.1^+^/C-(K)-DYK vector cloned *GAA* (NM_000152.5) open reading frame (ORF) was commercially obtained from Genscript (https://www.genscript.com). Cloned *GAA* ORF nucleotide sequence was 2,859 base pairs (bp) long, making a recombinant vector length of 8,249 bp, which was used for transfection and expression in mammalian cell culture. This clone was used as a wild-type positive control as well as a template for generating targeted point mutations using site directed mutagenesis.

### 2.2 Mutagenic primer design and synthesis

As per manufacturer’s instructions, 24–27 bp mutant primers with the desired nucleotide substitution in the center of the oligonucleotide sequence were designed and synthesized (Integrated DNA Technologies). Each primer was checked for the T_m_ (melting temperature), and secondary structure formation. Both the forward and reverse sequences of the mutant primers for each *GAA* variant, with a specific nucleotide substitution are listed in Supplementary Materials.

### 2.3 Synthesis of pcDNA3.1 cloned GAA targeted variants

GenEZ *GAA* (NM_000152.5) ORF cloned in pcDNA3.1^+^/C-(K)-DYK vector was used as a template. Following manufacturers’ instructions (Quikchange II XL site directed mutagenesis by Agilent Technologies), PCR reactions were performed and the mutant primer was extended to generate each targeted pcDNA3.1 cloned *GAA* point mutation. Mutant plasmid clones were transformed with XL10-Gold ultracompetent *E. coli* cells provided with the kit. Isolated colonies of specific mutant *GAA* clones were cultured, and plasmid was purified (Qiagen maxiprep kit). Subsequently, purified plasmid was sequenced to confirm the targeted nucleotide substitution.

### 2.4 HEK293 mammal cell culture-based expression and functional assay of mutant constructs

HEK293 cells were transfected with wild-type pcDNA3.1-*GAA* and with mutant constructs, using standard calcium phosphate-DNA precipitation methods. HEK293 cell culture plates (10 cm) were transfected with 10 µg of cloned *GAA* plasmid. Cultured cells were harvested after 48 h of transfection and were lysed by sonication. Each mutant and wild-type cell lysate was analyzed for protein concentration using a bicinchoninic acid (BCA) assay and volumes were adjusted for uniform protein concentrations. GAA enzyme activity was measured using artificial substrate 4-methylumbelliferyl-α–D glucopyranoside (4MUG; Sigma-Aldrich) and expressed as nM/hour/mg protein. For evaluation of each *GAA* variant, HEK293 cells with mock transfection (negative control) and cultures transfected with wild-type pcDNA3.1-*GAA* (positive control) were utilized as controls. Each variant was expressed in two separate HEK cell culture plates, and independently analyzed for GAA enzyme activity and protein characterization. Values reported in Table 3 are the average of these two independent experiments. Western blot analysis was also performed on cell lysate protein using 10% SDS-PAGE gels following standard protocols (Laemmli, 1970; Towbin et al., 1979). Mouse anti-human *GAA* antibody was used to visualize protein bands using a chemi-luminescence detection kit by GE Healthcare.

**TABLE 3:** GAA enzyme activity of each variant tested in the HEK293 cell-based functional analysis assay. Values in parenthesis are average of 2 experiments.

Patient	Variant	% Residual GAA activity
1	c.316C >T (p.Arg106Cys)	11.05, 10.35 (10.7)
2	c.1103G >A (p.Gly368Asp)	2.6, 2.9 (2.8)
3	c.1721T >C (p.Leu574Pro)	Not detectable
4	c.664G >A (p.Val222Met)	11.4, 12.1 (11.7)
	c.2450A >G (p.His817Arg)	Not detectable
5	c.1048G >A (p. Val350Met)	1.8, 1.2 (1.5)
6	c.1378G >A (p.Glu460Lys)	18.9, 16 (17.5)
7	c.1123C >T (p. Arg375Cys)	10.8, 12.5 (11.7)

## 3 Results

### 3.1 Functional assay validation

The known pathogenic *GAA* variants used for validation of the functional assay were found to have reduced or deficient levels of GAA activity (range = 0%–11% of normal) as shown in Table 1. In contrast, the known likely benign/benign *GAA* variants were found to have a GAA enzyme activity range of 54%–100% of normal. The well-known pseudodeficiency variant (c.1726G >A; p. Gly576Ser) had a GAA enzyme activity of 17% of normal, falling slightly above the highest enzyme activity of a pathogenic control (11%) and well below the lowest level of GAA activity seen in a benign control (54%) (Table 1).

### 3.2 Identification and analysis of variants among patients

#### 3.2.1 Patient #1

Clinical Presentation: The patient is a 6-month-old Caucasian male diagnosed with LOPD after positive NBS. Average GAA enzyme activity on dried blood spot (DBS) was 1.59 µmol/hr, reported as 13.9% of daily mean activity (normal range >15%). Confirmatory enzyme testing in leukocytes revealed decreased GAA activity at 5.1 nmol/hr/mg (reference range: 23.1-232.0). *GAA* sequencing revealed two variants, c.−32-13T >G (pathogenic, “late-onset” variant) and c.316C >T (p.Arg106Cys) reported as a VUS. Parental testing confirmed that these variants were in trans. Echocardiogram was negative for hypertrophic cardiomyopathy (HCM). The patient had overall normal serum biomarkers including CK, AST, and ALT and normal urine Glc_4_. He was meeting developmental milestones for age but performed slightly below average for age on standardized physical therapy assessments.

Results: pcDNA3.1 cloned *GAA* with c.316C >T (p.Arg106Cys) demonstrated reduced GAA enzyme activity at 10.7% of the wild-type positive control (Table 3). Protein characterization by western blot revealed faint precursor (110 kDa) and mature (76 kDa) GAA protein bands but with lower intensity compared to the positive control, indicating this variant may be impacting GAA protein stability and processing.

#### 3.2.2 Patient #2

Clinical Presentation: The patient is an 11-month-old Caucasian male diagnosed with LOPD after positive NBS. GAA enzyme on DBS was low at 0.42 µmol/L/hr (normal range ≥2.1). *GAA* sequencing revealed two variants, c.−32-13T >G (pathogenic, “late-onset” variant) and c.1103G>A (p.Gly368Asp), classified as a VUS. Parental testing confirmed that these variants were in trans. Echocardiogram was negative for HCM. Serum biomarkers (CK, AST, and ALT) remained persistently elevated, while Glc_4_ remained within normal limits. Although the patient met overall gross motor milestones, mild muscle weakness and postural concerns were noted on examination.

Results: pcDNA3.1 cloned *GAA* with c.1103G >A (p.Gly368Asp) demonstrated reduced GAA enzyme activity at 2.8% of the positive control (Table 3). Protein characterization by western blot revealed both precursor (110 KDa) and mature (76 KDa) GAA protein bands but with much lower intensity compared to the positive control, indicating this variant may be impacting GAA protein stability and processing.

#### 3.2.3 Patient #3

Clinical Presentation: The patient is a 13-month-old Caucasian male diagnosed with LOPD after positive NBS. GAA activity on DBS was low at 0.61 µmol/L/h (normal range ≥2.1) with a repeat GAA activity of 0.41 µmol/L/hr. Confirmatory enzyme testing in leukocytes revealed GAA activity of 0.8 µmol/L/hr. *GAA* sequencing revealed two variants, c.−32-13T>G (pathogenic, “late-onset” variant) and c.1721T >C (p.Leu574Pro). Parental testing confirmed that the variants were in trans. Echocardiogram was negative for HCM. Initial serum biomarkers were normal, but were trending upward and urine Glc_4_ remained within normal limits. He had mild postural concerns and signs of muscle weakness but overall met gross motor milestones appropriately.

Results: pcDNA3.1 cloned *GAA* with c.1721T>C (p.Leu574Pro) demonstrated complete loss of catalytic GAA activity (Table 3). Protein characterization by western blot revealed only the precursor (110 KDa) GAA protein band, with no visible mature protein band. Absence of the mature form of the GAA protein may be an indication of aberrant post-translational modification and localization.

#### 3.2.4 Patient #4

Clinical Presentation: The patient is a 15-month-old Caucasian male diagnosed with LOPD after positive NBS. GAA enzyme activity in DBS was 8.2% of normal (normal range: >15% of daily mean). GAA enzyme activity was repeated in blood at 6 months of age was found to be in the deficiency range seen in patients affected with Pompe disease (3.3 nmol/hr/mg protein; normal range = 23.1-232.0). He was reported to have two *GAA* VUS in trans: c.664G >A (p.Val222Met) inherited from his father and c.2450A >G (p.His817Arg) inherited from his mother. The patient’s parents had blood GAA enzyme analysis, and each was found to have enzyme levels in the indeterminate range, as is often observed in carriers: mother: 6.60 pmol/punch/hr and father: 7.70 pmol/punch/hr (affected range: ≤3.88; normal range: >10.88). Echocardiogram was negative for HCM. Although CK was initially normal, it was elevated at age 6 months, but by 15 months it returned to normal. Glc_4_ was elevated at 2 weeks of life but was within normal limits at age 15 months when adjusted for age. He was appropriately meeting developmental milestones. On physical therapy evaluation, he had age-appropriate gross motor skills and some mild postural concerns.

Results: pcDNA3.1 cloned *GAA* with c.664G>A (p.Val222Met) demonstrated reduced GAA enzyme activity at 11.7% of the positive control (Table 3). Protein characterization by western blot revealed precursor (110 kDa) and mature (76 kDa) GAA protein bands but with lower intensity compared to the positive control, indicating that this variant may be impacting functionally active GAA protein levels.

The second variant identified in this patient, pcDNA3.1 cloned *GAA* with c.2450A >G (p.His817Arg), demonstrated complete loss of GAA activity. Protein characterization by western blot revealed only the precursor (110 KDa) GAA protein band, with no visible mature protein band. Absence of the mature form of the GAA protein may be an indication of aberrant post-translational modification and localization.

Patients 5, 6 and 7 were identified *via* NBS at external institutions and de-identified data were provided for analysis and review. One pathogenic variant and one VUS or novel variant were detected in trans in each patient. All three patients were ascertained through positive NBS for Pompe disease with significantly reduced GAA enzyme activity levels on blood samples (Table 2).

#### 3.2.5 Patient #5

GAA enzyme activity in blood was 1.01 pmol/punch/hr (affected range ≤3.88). The patient had one pathogenic frameshift variant (c.1589del, p.Glu530Glyfs*48) and a missense variant (c.1048G>A; p. Val350Met).

Results: pcDNA3.1 cloned *GAA* with c.1048G>A (p.Val350Met) demonstrated reduced GAA enzyme activity at 1.5% of the positive control (Table 3). Protein characterization by western blot revealed faint precursor (110 k Da) and mature (76 kDa) protein bands but with lower intensity compared to the positive control, indicating this variant may be impacting GAA protein stability and processing.

#### 3.2.6 Patient #6

GAA enzyme activity in blood was 2.9 nM/mg Pr/h (normal range: 6.7-21.7). The patient had one pathogenic variant (c.−32-13T >G) in combination with a VUS (c.1378G >A; p. Glu460Lys).

Results: pcDNA3.1 cloned *GAA* with c.1378G >A (p.Glu460Lys) demonstrated reduced GAA enzyme activity at 17.5% of the positive control (Table 3). Protein characterization by western blot revealed both precursor (110 kDa) and mature (76 kDa) GAA protein bands, but with lower intensity compared to the positive control, indicating this variant may be impacting GAA protein stability and processing.

#### 3.2.7 Patient #7

GAA enzyme activity in blood was 0.94 µmol/L/h (normal range ≥2.10). The patient had one pathogenic variant (c.−32-13T>G) in combination with a missense variant (c.1123C >T; p. Arg375Cys).

Results: pcDNA3.1 cloned *GAA* with c.1123C>T (p. Arg375Cys) demonstrated reduced GAA enzyme activity at 11.7% of the positive control (Table 3). Protein characterization by western blot revealed both precursor (110 kDa) and mature (76 kDa) GAA protein bands, indicating this variant may be impacting GAA protein stability and processing.

### 3.3 Classification of variants

Following the ACMG/AMP variant classification standards and rules, and ClinGen SVI guidance for functional studies (Brnich et al., 2019; Richards et al., 2015), we applied PS3 at the supporting level in the classification of the variants identified in Patients 3 and 4 (c.1721T >C and c.2450A >G). Both variants showed absent/undetectable GAA enzyme activity in our *in vitro* functional assay, and the presence of only inactive precursor protein bands on western blot analysis, indicating a failure of GAA protein processing to mature active forms. Despite this functional data, c.2450A >G (Patient 4) remains a VUS due to insufficient evidence.

The remaining six variants had detectable, but reduced GAA enzyme activity using our *in vitro* functional assay. Three variants (c.316C >T, c.1103G>A, and c.1048G >A) had enzyme activity below the threshold established by the pathogenic control variants (11%). Thus, we applied PS3 at the supporting level. All three variants were subsequently classified as likely pathogenic. The remaining three variants (c.664G >A, c.1378G >A, and c.1123C >T) had enzyme activity levels between the thresholds established during our functional assay validation (11%–54%); therefore, PS3 could not be applied in the classification of these variants at any strength level. Table 4 illustrates the final classifications for all interrogated variants based on functional analysis and additional evidence using criteria established by the ACMG/AMP guidelines where pathogenic criteria are weighted as very strong (PVS1), Strong (PS1-4), Moderate (PM1-6), or Supporting (PP1-5) and benign criteria are weighted as standalone (BA1), strong (BS1-4) or supporting (BP1-6).

**TABLE 4:** Evidence used for variant classification using ClinGen *GAA* VCEP specifications^2^.

Patient	Variant	*In vitro* GAA activity (% wildtype) (<11% = PS3_supporting)	Allele frequency (gnomAD) PM2_supporting/BS1	In trans with a pathogenic variant (PM3)	Different missense variant at same codon, classified as pathogenic (PM5)	REVEL score (PP3/BP4)	Clinical phenotype for pompe disease (PP4_Moderate)	Final classification
1	c.316C >T (p.Arg106Cys)	10.7% **PS3_supporting**	<0.001 in all continental populations **PM2_supporting**	c.−32-13T >G **PM3**	NA	0.566	**PP4_Moderate**	Likely pathogenic (No conflict)
2	c.1103G >A (p.Gly368Asp)	2.8% **PS3_supporting**	Absent **PM2_supporting**	c.−32-13T >G **PM3**	NA	0.647	**PP4_Moderate**	Likely pathogenic (No conflict)
3	c.1721T >C (p.Leu574Pro)	Not detectable **PS3_supporting**	Absent **PM2_supporting**	c.−32-13T >G **PM3**	NA	0.958 **PP3**	**PP4_Moderate**	Likely pathogenic (No conflict)
4	c.664G >A (p.Val222Met)	11.7%	>0.005 (South Asians) BS1	NA	NA	0.446 **BP4**	**PP4_Moderate**	VUS (Evidence in conflict)
	c.2450A >G (p.His817Arg)	Not detectable **PS3_supporting**	Absent **PM2_supporting**	NA	NA	0.926 **PP3**	**PP4_Moderate**	VUS (Insufficient Evidence)
5	c.1048G >A (p. Val350Met)	1.5% **PS3_supporting**	0.00022 **PM2_supporting**	p.Glu530Glyfs*48 **PM3**	NA	0.878 **PP3**	**PP4_Moderate**	Likely Pathogenic (No conflict)
6	c.1378G >A (p.Glu460Lys)	17.5%	0.00002 **PM2_supporting**	c.−32-13T >G **PM3**	NA	0.546	**PP4_Moderate**	VUS (Evidence in conflict)
7	c.1123C >T (p.Arg375Cys)	11.7%	0.00004 **PM2_supporting**	c.−32-13T >G **PM3**	c.1124G >T (p.Arg375Leu) **PM5**	0.9539 **PP3**	**PP4_Moderate**	Likely Pathogenic (No conflict)

1 Sequence Variant Interpretation Working Group (SVI) updates - ClinGen | Clinical Genome Resource. https://clinicalgenome.org/tools/educational-resources/materials/sequence-variant-interpretation-working-group-svi-updates/.

2 ClinGen Lysosomal Storage Disorders Expert Panel Specifications to the ACMG/AMP Variant Interpretation Guidelines Version 2. https://www.clinicalgenome.org/affiliation/50009/docs/assertion-criteriaforthemostrecentversion.

## 4 Discussion

The utility of NBS for Pompe disease in the US has been demonstrated with the development of successful screening programs in many states resulting in significant improvements in clinical outcomes for IOPD (Li et al., 2021), yet there remains a challenge in appropriately diagnosing and managing patients with LOPD. This challenge is critically important to address because of the high number of patients with suspected LOPD ascertained *via* NBS (the vast majority of which do not have a phenotype in the neonatal period) and the large number of these infants with a genotype that includes a VUS or novel variant (Burton et al., 2017b; Klug et al., 2020). GAA enzyme analysis followed by *GAA* sequencing is critical for confirmation of a diagnosis of Pompe disease, and more specifically, for discernment between IOPD, LOPD, and pseudodeficiency alleles or carriers. The presence of cardiomyopathy or cardiomegaly within the first year of life is a clear distinguishing feature between IOPD and LOPD. The latter represents the majority of cases identified *via* NBS and over time, some of these infants have developed features of Pompe disease and have been initiated on ERT (Burton et al., 2017b; Huggins et al., 2022). Unclear diagnoses lead to uncertainty regarding disease management. Consequently, this could lead to unnecessary initiation or delay in initiation of ERT and anxiety for parents and caregivers. With more states adding Pompe disease to their NBS panels, the need for robust lines of evidence for *GAA* variant classification is more urgent than ever.

Although *in vitro* expression systems do not mimic nor represent the *in vivo* genetic complexity and human physiologic background, we demonstrate the utility of HEK293 cells in GAA expression systems. Expanding on prior work (Kroos et al., 2012), we have utilized ClinGen guidance and have established functional assay control ranges using known pathogenic (*n* = 7), likely benign/benign (*n* = 4), and a pseudodeficiency (*n* = 1) variant. Our GAA expression system for measuring GAA enzyme activity combined with immunoblotting for GAA protein analysis has been effective in characterizing the behavior of specific *GAA* variants *in vitro*. In addition, recommendations from the ClinGen Variant Curation Expert Panel (VCEP) for *GAA,* which consists of a team of highly experienced clinicians and scientists in the field of Pompe disease, were utilized to further tailor the ultimate classification of the *GAA* variants examined.

The application of PS3_supporting based on our novel functional data was critical to the classifications of c.316C >T and c.1103G >A as likely pathogenic. The clinical and enzymatic data available for both probands allowed for the application of PP4_moderate, and each of these variants was detected in trans with a known pathogenic variant (PM3). The frequency of these variants in the general population met the threshold for the application of PM2_supporting; however, the REVEL score did not meet the threshold established by the ClinGen GAA VCEP (>0.7), and PP3 could not be applied. Without the novel functional data, those variants would remain classified as VUS.

Additional clinical details and publicly available information was critical for the classification of variants examined by this study (Table 4). Despite having an *in vitro* enzyme activity level (11.7%) just above the set threshold (<11%), c.1123C >T can be classified as likely pathogenic based on other evidence, including the classification of a different missense change at the same amino acid position c.1124G>T; p.Arg375Leu as pathogenic (six independent likely pathogenic/pathogenic ClinVar submissions), allowing the PM5 criterion to be applied. While our functional data supports the classification of c.1721T >C and c.1048G >A as deleterious variants with *in vitro* enzyme activity levels of <2%, these variants could be classified as likely pathogenic using only the patient clinical data and additional publicly available information such as the variant allele frequency in the general population (gnomAD) and *in silico* prediction algorithms (REVEL score).

The classification of two variants, c.664G >A and c.2450G >A, is complicated by the fact that they were observed in trans in the same patient (Patient 4). Although our functional data suggests that c.2450G >A is clearly deleterious (no residual enzyme activity), the lack of additional evidence at the moderate strength level means that this variant remains of uncertain significance. The in trans variant in Patient 4, c.664G >A, had an *in vitro* enzyme activity level in the indeterminate range, but has an allele frequency high enough in the South Asian population for BS1 to apply, and a REVEL score that meets the threshold for the application of BP4 as evidence toward a benign classification. The combination of a strong and a supporting piece of evidence can result in a likely benign classification. This variant has not been curated by the ClinGen *GAA* VCEP, and is classified as likely benign/benign by five independent ClinVar submitters. Given the deficient enzyme activity observed in Patient 4, and the indeterminate (carrier range) enzyme activity level of the heterozygous father, an impact on enzyme activity cannot be excluded. This variant may cause reduced enzyme activity (*in vitro* and/or *in vivo*), but not in the range typically observed for truly pathogenic alleles. Both of the variants observed in Patient 4 remain classified as VUS.

One additional variant interrogated by our functional assay remains classified as a VUS: c.1378G >A. While this patient (Patient 6) meets the clinical and *in vivo* enzymatic criteria for the application of PP4_moderate, and a known pathogenic variant was detected and proven to be in trans (PM3), our functional assay showed an *in vitro* enzyme activity in the indeterminate range (17.5%), and the REVEL score (0.546) does not meet the threshold for the application of PP3. In fact, the REVEL score is just above the threshold for the application of BP4 (<0.5) in favor of a benign classification. This variant remains classified as a VUS due to conflicting evidence.

In order to increase the strength at which the PS3/BS3 criteria can be applied using data from our assay, additional validation controls are needed. The ability to apply PS3/BS3 at the moderate level is especially important for the classification of rare or novel variants that have not been previously reported in the literature. Although we chose a variety of known pathogenic *GAA* variants (2 nonsense and 5 missense) as controls in order to cover a broader spectrum of residual GAA enzyme activity levels, there still remains a gap in residual enzyme activity levels in the indeterminate range (11%–54% of wild-type control) in our assay that presents a challenge for variant classification. Further work is needed to evaluate additional milder missense and splice site variants, including variants that have been exclusively reported in LOPD, that may fall within this enzyme activity range to aid in classification. We acknowledge this conservative approach in establishing the threshold used for the application of PS3_supporting (<11%) was influenced by the pathogenic variants chosen as controls for functional assay validation. This threshold may change as additional known pathogenic variants are assayed. Future data may indicate that a threshold value for the application of PS3_moderate may be more stringent than for the application of PS3_supporting, but further validation of the assay using additional pathogenic and benign controls is necessary and is currently underway. Three variants’ *in vitro* enzyme activity levels fell in the indeterminate range; we were therefore unable to apply the PS3_supporting criteria to aid in classification of these three variants: c.664G >A, c.1123C >T, and c.1378G >A. If our functional study had included the additional controls necessary for the application of PS3_moderate, we may have been able to reclassify c.2450A >G as likely pathogenic.

We have successfully interrogated eight variants identified through NBS for Pompe disease using our *in vitro* expression and functional analysis model using HEK293 cell lines and site-directed mutagenesis. Our assay was validated using 11 controls (7 pathogenic and 4 likely benign/benign variants) plus a pseudodeficiency variant. Using our assay, we were able to provide critical evidence to classify 2 variants as likely pathogenic, and supporting evidence for 2 additional variants. To date, our focus has been on missense variants; however, this site-directed mutagenesis system can be used for other variant types identified in *GAA*. Careful consideration of clinical disease phenotype and presentation of each individual will remain crucial to accurate diagnosis. We will continue to refine our robust and reproducible *in vitro* functional analysis system which will allow us to continue to provide critical information to aid in the classification of many VUS and novel variants in *GAA*. This work aims to fill the growing unmet need for novel variant classification and molecular diagnostic confirmation for patients with positive NBS for Pompe disease.

## Data Availability

The original contributions presented in the study are included in the article/Supplementary Material, further inquiries can be directed to the corresponding author.

## Footnotes

1 Sequence Variant Interpretation Working Group (SVI) updates - ClinGen | Clinical Genome Resource. https://clinicalgenome.org/tools/educational-resources/materials/sequence-variant-interpretation-working-group-svi-updates/.

2
*ClinGen Lysosomal Storage Disorders Expert Panel Specifications to the ACMG/AMP Variant Interpretation Guidelines Version 2*. https://www.clinicalgenome.org/affiliation/50009/docs/assertion-criteriaforthemostrecentversion.
